# Chapter 4.5: New Proposed Treatments for Pelvic Organ Prolapse

**DOI:** 10.1007/s00192-025-06450-1

**Published:** 2025-12-01

**Authors:** Marianna Alperin, Fatima F. Fitz, Caroline E. Gargett, Zeliha Guler, Cheryl B. Iglesia, Cassandra K. Kisby, Svjetlana Lozo, Valentin Manriquez, Srikala Prasad, Carolyn W. Swenson, Julie A. Suyama, Maria A. T. Bortolini

**Affiliations:** 1 Department of Obstetrics, Gynecology, and Reproductive Sciences, Division of Urogynecology and Reconstructive Pelvic Surgery, University of California San Diego, San Diego, CA USA; 2 Sanford Consortium for Regenerative Medicine, 2880 Torrey Pines Scenic Drive, La Jolla, CA 92037 USA; 3 Department of Gynecology, Sector of Urogynecology and Pelvic Floor Reconstructive Surgery, Federal University of São Paulo, São Paulo, Brazil; 4 The Ritchie Centre, Hudson Institute of Medical Research, Melbourne, VIC 3168 Australia; 5 Department of Obstetrics and Gynaecology, Monash University, Melbourne, VIC 3800 Australia; 6 Department of Obstetrics and Gynecology, Amsterdam UMC, Amsterdam, the Netherlands; 7 Reproductive Biology Laboratory, Amsterdam UMC, Amsterdam, the Netherlands; 8 Amsterdam Reproduction and Development Research Institute, Amsterdam, the Netherlands; 9 Section of Female Pelvic Medicine and Reconstructive Surgery, MedStar Washington Hospital Center, Washington, DC USA; 10 Georgetown University School of Medicine, Washington, DC USA; 11 Department of Ob/Gyn, Division of Urogynecology, Duke Health, Duke University School of Medicine, Durham, NC, USA; 12 Columbia University Irving Medical Center, New York, USA; 13 Pelvic Floor Unit. Clinical, Hospital University of Chile, Región Metropolitana, Chile; 14 Department of Urology, Government Kilpauk Medical College, Chennai, Tamilnadu India; 15 Division of Urogynecology & Reconstructive Pelvic Surgery, Department of Obstetrics and Gynecology, University of Utah, Salt Lake City, UT USA; 16 Department of Obstetrics & Gynecology, Division of Urogynecology & Reconstructive Pelvic Surgery, Virginia Commonwealth University Health, Richmond, VA USA

**Keywords:** Pelvic organ prolapse, Basic science, Translational research, Novel treatments, Regenerative medicine

## Abstract

Pelvic organ prolapse (POP) is a morbid and costly condition that affects millions of women worldwide. Given the shortcommings of the current treatments, novel preventative and therapeutic approaches are needed. This manuscript is part of the International Urogynaecologogy Consultation (IUC) on pelvic organ prolapse (POP) chapter four on new and novel treatments for pelvic organ prolapse. The current expert narrative review (1) highlights the rationale for novel treatments for POP; (2) summarizes the exisitng mechanistic insights into physiologic alterations needed to inform the development of novel preventative and therapeutic strategies for POP; (3) reviews relevant modern tools that can help establish causal relationships between epidemiologic risk factors and POP pathogenesis; (4) describes prevention-focused interventions and advancements in treatment-focused interventions to date; and (4) emphasizes requirements for responsible translation of discoveries into novel treatments and safe incorporation of new treatments into clinical practice. Importantly, the review underscores the need for multidisciplinary adequately funded research and training programs as an absolute prerequisite for enabling a long overdue shift in clinical paradigm—instead of relying on delayed compensatory “one-size-fits-all” treatments that do not address the underlying pathophysiology, the focus should be on preventing or mitigating POP through personalized medicine approaches supported by team science.

## Introduction

The present report, focused on new therapeutic and preventative approaches for pelvic organ prolapse (POP), is a part of the International Urogynecology Consultation (IUC), sponsored by the International Urogynecological Association (IUGA). It is the last of five reviews in the fourth chapter, with the others dedicated to: (4.1) outcomes of POP surgery; (4.2) impact of surgical treatment of POP on pelvic floor symptoms; (4.3) management of complications of POP surgery; and (4.4) financial costs of POP surgery. Given the focus on new proposed treatments for POP, a systematic review was not possible, thus narrative review was completed by the committee.

## Materials and Methods

The chair of chapter 4.5 was selected by the IUC steering committee with approval of the IUGA executive committee. The writing group was selected through the IUC steering committee after a competitive application process and by invitation from the chair. In addition to the chair and the IUC steering committee member, this narrative review was completed by 10 international clinical and basic science experts in POP. Given the focus of chapter 4.5 on the evolution of novel treatements for POP, systematic review was not applicable. Therefore, the outline and the overarching content of this narrative review, as well as a list of search terms were determined by expert consensus. This information was presented at the IUGA 2022 annual scientific meeting for input from the general membership. No additional content or search terms were suggested by the attendees of the meeting. The following databases were searched: PubMed, Embase and LILAC’s, and the articles captured from inception until September 2023. Owing to the unique nature of this topic, narrative review was employed obviating the need for grading. Therefore, two senior members of the group reviewed all of the manuscripts and selected those for inclusion onto the final document. Cross referencing was done where appropriate. The writing group was divided into four subgroups based on specific expertise and experience of the individual members. The subgroups focused on the following rubrics: (1) scientific rationale for evolution of novel treatments; (2) prevention-focused interventions; (3) treatment-focused interventions; and (4) safe incorporation of new treatments into clinical practice. Selected members of the group worked together on the recommendations for future directions. The chair edited and put together the versions of the above rubrics into a single cohesive review, the draft of which was sent to all writing group members for further edits. Upon incorporation of everyone’s feedback, the draft was sent for the established IUC approval process, after which it was submitted to the International Urogynecology Journal for peer-review and publication.

## Rationale for Evolution of Novel Treatments

Pelvic organ prolapse (POP) is an ancient problem that has negatively impacted women from different racial, ethnic, and cultural backgrounds for thousands of years. The high prevalence of this morbid condition is related to the main epidemiological risk factors associated with POP; vaginal childbirth and aging, experienced by many women over their lifetime. Despite its longstanding history and high prevalence, the pathogenesis of POP has not been fully elucidated to date, impairing development of effective preventative strategies. Significant long-standing knowledge gaps also limit our ability to advance therapeutic options for POP. Existing treatments for symptomatic POP are limited to pelvic floor rehabilitation that has minimal impact on POP distal to the introitus—a point when POP becomes bothersome; pessaries that are associated with various complications and have not changed conceptually since 1800 BC; and surgeries that are plagued by either adverse outcomes or high failure rates. The extraordinary prevalence of POP, the negative impact of this condition on quality of life, and high associated costs to the individuals and societies are in stark contrast with the lack of substantive changes in POP treatments for multiple decades. Thus, there is an urgent need to develop novel therapeutic approaches. Furthermore, as the number of women suffering from POP continues to increase due to an aging population, effective preventative strategies are needed more than ever.

### Limitations of the Existing Treatments

Most of the current treatments for POP are delayed and compensatory, as they do not address the underlying pathophysiology. Together with the associated complications, the above significantly limits the effectiveness of the available interventions. While conservative measures, such as pessaries, are accepted by many patients with POP, with very few contraindications to their use, only 14% of women continue pessary use long-term due to the associated vaginal dysbiosis, discharge, foul odor, vaginal epithelial abnormalities, and bleeding [1]. Furthermore, with little to no innovation in pessary design for decades, many women discontinue its use due to difficulties with self-management, need to remove for intercourse, or increased stress urinary incontinence with prolapse pessaries. Poor long-term adherence to pessary use leads to a high rate of surgical interventions for POP [2, 3].

With respect to surgical POP management, native tissue repairs are associated with high failure rate, while mesh-augmented abdominal repair is more invasive and has a higher rate of perioperative complications [4]. While systematic reviews of transvaginal POP repairs with permanent mesh demonstrate lower objective and subjective POP recurrence compared to native tissue vaginal procedures [5], the high rate of mesh-related complications led to the abandonment of this approach throughout most of the world. Unfortunately, the absorbable meshes or biological grafts do not improve surgical outcomes compared to the native tissue POP repair [5, 6]. In addition, patient centered outcomes following transvaginal and abdominal surgeries for apical prolapse reveal > 15% rate of severe complications and related regret and low quality of life based on patient-perspectives in adverse event reporting at 3 years follow-up [7].

### Lack of Preventative Strategies

Given the high prevalence of POP and suboptimal treatments, it is imperative to develop preventative strategies for this common condition. While maternal injury arising from vaginal birth is considered a major underlying risk factor for developing subsequent POP, there is a lack of consistent and effective preventative strategies that could be applied during pregnancy and the peripartum period [8], and subsequently as women age. Neither is it an appropriate strategy for all women to undergo cesarian delivery, when most do not develop POP, as with this strategy many more women would be exposed to unwarranted surgical risks [9]. It is therefore important to accurately identify women at highest risk of developing POP antepartum to enable individualized counseling regarding the mode of delivery. Maternal birth injury can occur to one or more of the pelvic soft tissues—and in any combination, complicating the development of preventative strategies. While many studies describing alterations in pelvic supportive ligaments, vagina, and pelvic floor muscles exist, little attention has been paid to the importance of superficial perineal muscle and perineal body injuries during vaginal birth that lead to an enlarged urogenital hiatus and how these can be prevented [10]. One suggestion is to measure the urogenital hiatus and fetal head size late in pregnancy [11] using transvaginal ultrasound or MRI for predicting pelvic soft tissue injury and making decisions regarding the safest birthing process. However, changing established obstetrical practices remains very challenging. For example, despite lack of significant differences in neonatal outcomes but the undeniable evidence that forceps-assisted vaginal deliveries confer greater risk of POP and other pelvic floor disorders compared to vacuum-assisted vaginal deliveries [12, 13], forceps continue to be widely deployed in clinical practices across the globe.

The long latency period between childbirth (the inciting event) and the progression to symptomatic POP has made it difficult to identify the actual cause and therefore develop effective preventative strategies. The cellular and molecular mechanisms causing major changes in the biomechanical properties of the vagina-supportive tissue complex and pelvic floor muscles during pregnancy and why they are inadequate in some pregnant women hampers the development of strategies to overcome such deficiencies to prevent maternal birth trauma. Following childbearing, multiple intervening factors, detailed below, further exacerbate and accelerate the development of POP. It is clear that an effective screening strategy is required to identify women at high risk of maternal birth injury to enable appropriate preventative interventions [14].

### Biochemical Causes of POP and Secondary Prevention

Lack of secondary prevention options arise from a lack of scientific knowledge of residual alterations at the cellular, microscopic, and nanoscopic level in grossly “healed” pelvic soft tissues following vaginal birth trauma. Studies to better understand the biochemical processes of injury and healing are needed to identify targets before strategies can be applied for POP prevention. Secondary prevention is a pragmatic strategy that has been used for other chronic medical conditions, such as those predisposed to cardiovascular disease who are monitored by measuring blood pressure and cholesterol levels followed by prescribing of appropriate correcting drugs, such as statins and antihypertensives [14]. For tissue injury, regenerative medicine approaches should be considered as an alternative to the existing compensatory treatments. A cell-based therapy is one potential secondary prevention strategy, in particular mesenchymal stem cell (MSC) based therapies as MSC promote endogenous stem cell recruitment in soft tissue injuries, dampen inflammation, promote angiogenesis, and reduce fibrosis [15]. Another promising approach is the use of acellular biomaterial scaffolds to promote host cell infiltration and endogenous regeneration. For detailed review of the state-of-the-art of regenerative medicine for PFDs, please refer to a recent publication by Henderson et al. [16].

## Mechanistic Insights into Physiologic Alterations Needed to Inform the Development of Novel Preventative and Therapeutic Strategies for POP

While strides have been made in understanding the contributions of various risk factors, described below, to the pathophysiology of POP, accurate individualized risk assessment and preventative strategies continue to be severely limited because: (1) the mechanisms of pelvic floor dysfunction that leads to the development of POP have not been fully elucidated and (2) the biological factors that segregate women who do not develop POP despite exposure to known epidemiological risk factors from those who do remain unknown.

One of the leading risk factors for POP development later in life is vaginal delivery. The magnitude of non-pathological phenotypic transformations that occur in pregnancy throughout a woman’s body and specifically in the pelvic organs and their supportive structures is unmatched. The main purpose of antepartum adaptations in the pelvis is to enable childbirth. Similarly to the competing demands of bipedalism and childbirth imposed on the bony pelvis [17], pregnancy-induced alterations in the biochemical, structural, and mechanical properties of pelvic soft tissues necessary to achieve fetal delivery are potentially detrimental to the supportive function of the female pelvic floor. Furthermore, antepartum changes that persist after vaginal delivery, such as loss of vaginal angulation relative to the levator plate due to decreased stiffness of vaginal supportive structures in pregnancy [18, 19], likely predispose pelvic floor soft tissues to insidious deterioration and eventual dysfunction, leading to the development of symptomatic POP decades later. Understanding functionally relevant changes that occur at tissue and cellular levels in the pelvic floor supportive structures due to pregnancy and childbirth is essential for identifying effective preventative and treatment strategies for POP. Better understanding these factors may enable individualized counseling of women regarding their risks and suitability for various treatment options. The same holds true for all other predisposing, inciting, and intervening events that lead to the development of POP along a woman’s lifespan.

### Understanding the Biochemical Physiology of POP

We live in the era of ever-expanding personalized medicine approaches, which use individual’s genomic, proteomic, and metabolomic phenotypes as biomarkers to make treatment decisions, in various fields. Unfortunately, such approaches have not been deployed in clinical care of women with POP to date. A recent study highlights the importance of deeper phenotyping to identify novel therapeutic targets to counteract POP [20]. The uterosacral ligaments—important apical supportive structures—were procured from women with and without (control) POP and analyzed histologically using a novel Pelvic Organ Prolapse Histologic Quantification (POP-HQ) system. Using nine morphologic parameters and unsupervised principal component analysis, three distinct histologic phenotypes were identified in the POP group: POP-V, characterized by vascular alterations; POP-A, with substantial adipose infiltration; and POP-I, with significant increase in inflammatory infiltrate. POP-I phenotype was the strongest histologic predictor of POP after adjusting for other risk factors, as it diverged the most from the controls, highlighting the importance of *downregulating chronic inflammatory responses*. Another group in Korea demonstrated marked oxidative DNA damage, lipid peroxidation, and increased mitochondrial apoptosis in the uterosacral ligament of women with stages III & IV POP compared to the controls with stage 0 or I pelvic organ support. Thus, *approaches that modulate oxidative stress* should be investigated in the context of POP [21]. Overall, these tissue level findings provide novel therapeutic targets for possible secondary preventative measures to counteract detrimental changes in the important pelvic supportive structures. A recent review by Wu X et al. outlines pelvic tissue-level findings in women with vs without prolapse that point to various potential molecular biomarkers and therapeutic targets to counteract POP [22].

Direct tissue studies are difficult to execute in living women; thus, in vivo investigations in preclinical animal models [23, 24] and in vitro animal experiments are invaluable for gaining mechanistic insights that are needed for the development of scientifically rational preventative and therapeutic options. Murine models that can be genetically manipulated are important for establishing cause–effect relationships. Genetic perturbations (knocking out (KO) genes) that preclude proper assembly of the main fibrillar extracellular matrix (ECM) components, such as collagen and elastin, lead to POP in these animals, underscoring the important role of connective tissues in pelvic organ support [24–27]. The development of POP in the KO animals provides a mechanistic link between morphometric, ultrastructure, and biochemical alterations of connective tissues observed in women with POP [28–33]. Studies mimicking strains associated with parturition using vaginal balloon distention in mice resulted in increased vaginal matrix metalloproteases MMP-2 and MMP-9 activity, regulators of collagen synthesis and breakdown [34]. This was accompanied by visible fragmented and disrupted elastic fibers compared to the well-organized elastin fibrills in the vagina of animals in the sham group. The tropoelastin, a monomer that is assembled and organized into mature elastic fibers under the regulation of fibulin-5, also increased in pregnant animals in the distention group. Analogous distention in fibulin-5-deficient (*Fbln5*^−/−^) mice with defective elastic fiber synthesis and assembly induced accelerated POP compared to the wild type mice, which never recovered. Taken together, these data indicate that mechanical strains associated with parturition stimulate vaginal protease activity but also increase synthesis of proteins important for elastic fiber assembly. The finding that distention results in accelerated POP in *Fbln5*^−/−^ animals, but not in wild type mice, indicates that elastic fiber synthesis is crucial for recovery of the vaginal biochemical and mechanical properties. Importantly, deletion of MMP-9 in *Fbln5*^−/−^ resulted in increased elastic fiber density and improved collagen fibrils, in turn, significantly attenuating POP in these animals [35]. Interestingly, significantly increased levels of MMP-9 were identified in vaginal biopsies procured from pre- and postmenopausal women with POP compared to age-matched controls without POP [35]. Similarly, MMP-2 and MMP-12 activity was increased, while activity of tissue inhibitors of metalloproteinase that neutralized MMPs was decreased in anterior vaginal wall biopsies of premenopausal women with compared to without POP [36]. Together, these findings indicate that *therapeutics that block matrix protease activity hold promise for promoting proper healing and preventing POP development and mitigating POP progression in women*.

### Secondary Prevention Measures: Translation of Mechanistic Insights into Better Treatments and Effective Preventative Strategies for POP

MMPs are known to degrade vaginal FBLN5 in the rat model of simulated birth injury. Importantly, the above mechanistic insights led to the application of actinonin, a broad-spectrum MMP inhibitor, to block injury-mediated degradation of vaginal FBLN5 in nonpregnant [37] and pregnant [38] rat models of simulated birth injury. Treatment with actinonin mitigated the degradation of vaginal FBLN5 and ameliorated the negative effects of injury on vaginal biomechanical properties compared to the untreated animals. The above provides invaluable preclinical data to pursue human trials to evaluate *actinonin as a novel preventative strategy for POP in women*. Animal models of aging also demonstrate aberrations in the major extracellular matrix components known to impact biomechanical properties of pelvic tissues [39].

In other fields of medicine, discoveries of disease pathogenesis have provided the rationale for developing successful treatments and effective preventative strategies. For example, many disorders and diseases, like POP, are associated with aging. At the cellular level, aging manifests as cellular senescence, a process involving loss of telomeres which leads to irreversible cell cycle arrest [40]. Aging-associated tissue phenotype can also result from previous injury as in maternal birth trauma. Prematurely senescent cells persist, accumulate, and secrete SASP (senescence-associated secretory phenotype) molecules, which promote exhaustion of cellular progenitors, mitochondrial dysfunction, chronic inflammation (inflammaging), tissue fibrosis, and alterations in the metabolome, all of which, in turn, increase the risk of developing symptomatic POP. Cells with SASP secrete ECM proteases and remodeling factors, tissue inhibitors of matrix metalloproteinases (TIMPs), reactive oxygen species, cytokines, chemokines, and bioactive lipids, which create a toxic environment for remaining healthy cells locally and systemically. The transient SASP of senescent placental cells leads to a pro-inflammatory state that promotes parturition and clearance of any residual senescent cells [41]. The combination of concomitant injury to the pelvic support structures due to persistent SASP could be a major factor leading to the development of POP. As stated later, aging is associated with accumulation of senescent cells due to reduced immune clearance and maybe a potential therapeutic target for prevention and treatment of POP. For example, investigators at the Mayo clinic are currently conducting a phase 2 randomized clinical trial (AFFIRM – Alleviation of Frailty, Inflammation, and Related Measures, NCT03430037) of a single senolytic, fisetin, in women > 70 years with gait disturbance to evaluate whether targeting inflammation will reduce markers of insulin resistance, bone resorption and physical dysfunction. Depending on the outcome of this and numerous other clinical trials [40], senolytics could be considered for preventing or treating POP. They will first need evaluation in relevant preclinical models.

Another finding is that untoward changes in biochemical and biomechanical properties of pelvic connective tissues and skeletal muscles predispose one to POP with progressive negative alterations following POP development contributing to the high rates of POP recurrence following native tissue repairs. In other words, vaginal tissue of patients with POP becomes increasingly stiff leading to worsening POP. Surgery to correct POP can lead to increased stiffness through improper healing (as discussed above) predisposing to POP recurrence or de novo POP in another compartment. Studies focused on dissecting this vicious cycle have demonstrated that increased mechanical stretch and higher vaginal stiffness associated with POP negatively impact the function of vaginal fibroblasts [42], integral for the ECM homeostasis, further exacerbating POP progression. The current clinical paradigm discourages interventions for asymptomatic POP (probably when tissues are less stiff); however, the above findings challenge this approach. Early interventions might be beneficial by ameliorating continued mechanical changes in the prolapsed tissues that negatively impact function of the endogenous fibroblasts [43].

Understanding the physiology of the biochemical changes leading to altered healing following birth trauma and with aging will allow the development of secondary prevention strategies by intervening at the cellular and biochemical level. Using therapeutics aimed at better recovery and delayed senescence of the tissues that support the pelvic organs may become potent preventative strategies both at the time of initial injury (child birth) and any following subsequent injury as involved with surgical intervention.

## Requirements for Translating Discoveries into Novel Treatments

**Fig. 1:**
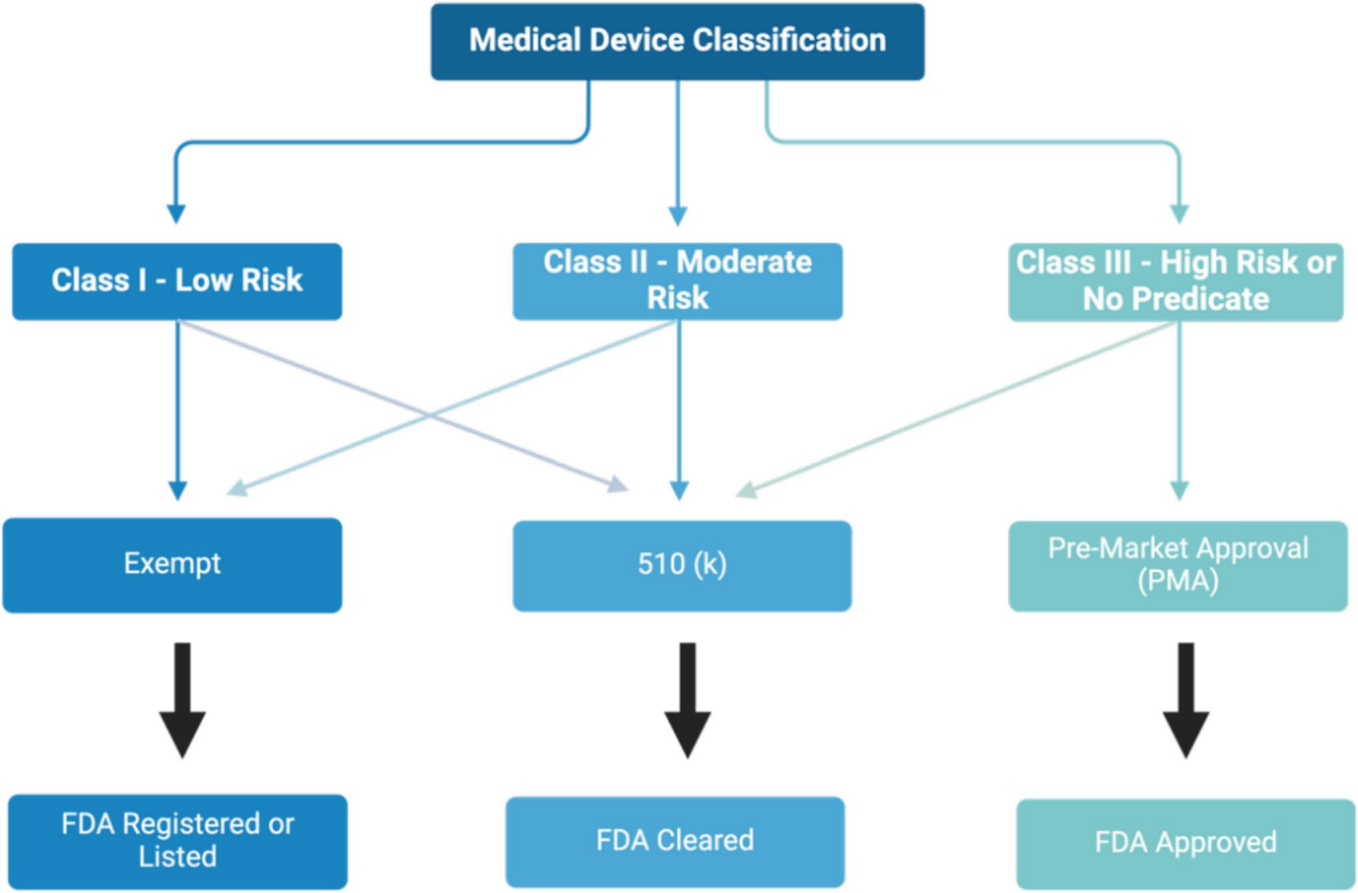
Flow chart of the registration, clearance, and approval of medical devices by the Food and Drug Administration (FDA) based on classifications utilized in the USA

Figure 1 shows the steps required to develop preventative approaches often used in other fields of medicine which could be adapted for birth trauma prevention and for progression to POP. Mechanistic studies required for the second step are in progress, step three requires the introduction of preventative measures and step 4 is assessing their effectiveness through randomized clinical trials and introduction of new practices into clinical management.

Basic science discoveries provide mechanistic insight into POP pathogenesis and identifies potentially new approaches for treating POP. However, a number of major steps are required in translating this basic research into a therapy for use in the clinic. In vitro assays and small animal models generate the initial proof-of-principle data. For example, design of new mesh materials matching the biomechanical properties of human vaginal tissue to prevent mesh exposure and erosions was first assessed in a rat abdominal hernia model [44]. It is recommended that such studies could then be followed by evaluation in large animal preclinical models closely replicating, as is possible, the clinical condition to be treated by such new therapeutic candidates [45]. For POP, these would use the vaginal site and similar surgical procedures used in women. The ovine model is relevant as sheep develop spontaneous acute POP associated with parturition [24]. Parous sheep show evidence of compromised biomechanical vaginal properties, which can be detected noninvasively by modified POP-Q measurements or intravaginal devices [46, 47]. Patent protection and engagement with regulatory authorities such as the Food and Drug Administration in the USA are essential for the development of protocols, standard operating procedures, good manufacturing practice, and meeting the required documentary evidence for obtaining regulatory approval for a clinical trial of any new device, as detailed below. Team-science collaborations are necessary to undertake clinical trials from phase 1 (safety and dosing), through phase 2 (efficacy and side effects), to phase 3 (comparison of the new intervention to an existing treatment, standard care, or placebo). The importance of well-designed clinical trials with consistent terminology and standardized outcomes [48] measured over biologically relevant time periods using adequate sample size has been stressed in Cochrane reviews on POP treatments [5]. Education of clinical teams in administering any new therapy or new procedure and development of clinical guidelines are also required.

For POP treatment, recent scientific discoveries suggest that cells, such as mesenchymal stem cells (MSC) or their derivatives, small membrane bound extracellular vesicles (sEVs or exosomes), or acellular biomaterials that modulate the foreign body response and promote tissue repair, could be suitable for clinical translation [49, 50]. The use of MSC and sEVs for wound healing and skin regeneration [51] or the use of tissue-specific extracellular matrix hydrogels for cardiac constructive remodeling [52] could be applied to injured pelvic supportive structures, delivered locally to the site of injury for potential secondary prevention of POP [53, 54]. However more research is required before such potential strategies can be deployed in women.

### Considerations in Developing New Devices for Treating POP

The impact of the host environment on efficacy and safety of a new therapy needs attention to identify suitable candidates [55]. It is first necessary to understand cellular and tissue-level changes associated with POP [56] and how these might impact the potential of a new therapeutic.

### Early Concept Therapeutics in POP Recognition and Treatment

One area of study involves the identification of pathological alterations to pelvic floor muscles following birth injury in women; atrophy, fibrosis, and inflammation. An area of primary consideration is how to identify those women predisposed to POP using objective diagnostic tests. A range of potential diagnostic approaches have been explored to objectively measure vaginal wall weakness in pregnant women and those suspected of having POP, including a tactile imaging probe for use in both antepartum period and following childbirth [57]. Various vaginal pressure sensor probes have also been developed to measure pelvic floor muscle strength [58] or vaginal integrity [59] without concurrently measuring intra-abdominal pressure. Shear wave elastography has been evaluated for assessing levator ani muscle elasticity in women with POP and to compare pelvic floor muscle recovery in women following vaginal birth and cesarean section [60, 61]. Similarly, vaginal elastometry using an automated hand-held intravaginal device assessed levator ani stiffness in postpartum women with obstetric anal sphincter injury [62]. A wearable intravaginal pressure sensor biofeedback device (Femfit®), has been developed to monitor daily activities that might impact POP, and to compare surgical outcomes of different POP reconstructive surgeries and for pelvic floor muscle training [63, 64]. Such devices provide biofeedback on daily activities in real time, but will need to consider cultural adaptations and the changing needs of women over the lifespan.

For treatment, in a proof-of-concept study in rats, a potential therapeutic skeletal muscle ECM hydrogel was injected immediately or 4 weeks after simulated birth injury into the damaged region. Alleviation of the damage was shown by enhanced muscle regeneration, reduced ECM remodeling, and modulation of the immune response [54].

Early studies on surgical therapeutics have recognized that native tissue repair often results in POP recurrence and there are problems associated with current implantable grafts to augment repairs. There is a need to modify biomaterial design and material type to take into account the ineffectiveness of current nonpermanent grafts and the negative sequelae of permanent transvaginal polypropylene mesh [65]. This has led to the pursuit of developing biodegradable polymeric materials for augmenting POP surgery using processes such as electrospinning, melt-electrowriting, and 3D printing to generate biomimetic implants [66, 67]. A variety of degradable biomaterials have been assessed in preclinical small and large animal models, including knitted monofilament poly-4-hydroxybutyrate (P4HB) in an ovine vaginal surgery model [68], electrospun poly(L-lactic acid)-*co*-poly ε-caprolactone (PLACL)/gelatin blend combined with human endometrial MSC in a mouse wound repair model [69], recombinant human tropoelastin:PCL electrospun yarns woven into a mesh in an ovine vaginal surgery model [70], and electrospun poly-L-lactic acid (PLA) and polyurethane (PU) into a rabbit abdominal hernia model [71]. Such biomaterials lend themselves to modifications to an estrogen-eluting or drug-eluting mesh to promote angiogenesis [72] and tissue ingrowth, particularly important in the use of degradable constructs. Rational mesh design that alters auxetic mesh geometries can overcome the pore collapse problem associated with polypropylene mesh [73]. This variety of degradable polymers with potential for augmenting future POP surgeries show the versatility of this approach to biomaterial design and modifications that can be tuned to the desired clinical condition. However, for translation of these new biomaterials, clinical trials will have to be conducted, regulatory approval obtained for medical devices (biomaterial only) and advanced medicinal products for tissue engineered cell therapy/biomaterial products, and registries established to monitor adverse outcomes [65]. Just as the biomaterial can be modified to develop improved treatments for augmenting POP surgery, the techniques used for generating implantable constructs can also be modified, particularly using tissue engineering principles of combining cells with biomaterials [74]. Endometrial, adipose tissue, and bone marrow MSC as well as skeletal muscle derived stem cells have been combined with both degradable and nondegradable biomaterials to make tissue engineering constructs and have been evaluated in a variety of preclinical models [74–76]. Endometrial MSC can be obtained in a relatively noninvasive manner from menstrual fluid or office-based endometrial biopsies. Other techniques involving adipose and bone marrow sources are more invasive requiring anesthesia, while muscle biopsies are painful and limited in size [77]. MSC secrete anti-inflammatory and immunomodulatory molecules, promote angiogenesis, and reduce fibrosis, making them an ideal cell type to incorporate into new biomaterial tissue engineering constructs being developed for treating and preventing POP [15]. Use of MSC in bioengineered constructs for POP requires a higher level of regulatory oversight to meet advanced medicinal product standards. They are expensive, particularly if an autologous approach is used. Problematically, allogeneic MSC that are massively cultured can quickly become senescent, one of the reasons for the underwhelming results in clinical trials for other conditions. These problems can be overcome by selecting the clonogenic perivascular MSC using specific markers, such as Sushi Domain Containing 2 (SUSD2) and small molecular weight inhibitors, e.g., A83-01, a TGFβ-receptor inhibitor, in culture expansion media to retain the MSC phenotype [15]. MSC extracellular vesicles or EVs, are membrane bound vesicles in the nanometer range that can also be used as they are the major component of the MSC secretome that targets pathophysiological processes involved in POP [78].

The advantage of sEVs is that they are acellular and can be used “off the shelf,” can be designed to contain desired molecules, and the regulatory burden, involved in studying and manufacturing, is much less. They would be simple to use postpartum for preventing POP. Another approach for generating bioengineered constructs for POP is to use 3D printed mesh with or without bioprinted MSC in a multi-step fabrication process [79]. 3D printed and melt electrowritten devices [80] offer the opportunity for precision therapeutics by the manufacture of patient-specific surgical mesh, as preoperative imaging can be integrated into computer assisted design (CAD) of personalized mesh designs [27, 66, 81]. These sophisticated approaches for developing new surgical constructs for treating and preventing POP are still in the proof-of-principle stage of development, offering new hope for improved treatments; however, they will require regulatory approval and clinical trials before they can be adopted into the clinic.

## Preclinical Animal Models for Assessing Disease Models and Outcomes

A variety of animal models are required to decipher the complex pathophysiology of POP and to investigate the efficacy of a range of new devices for diagnosing, treating, and preventing POP. Several models are usually needed to ensure all aspects including mechanism of action, efficacy, safety, and long-term monitoring are evaluated.

### POP Animal Disease Models

As noted previously, two knockout (KO) mouse models, the *Loxl1* KO and the *Fbln5* KO, have increased our understanding on the role of the ECM homeostasis in POP pathophysiology [27]. These models provide a mechanistic link between the connective tissue disorders in women, described below, and POP development, and demonstrate the importance of elastin biosynthesis and metabolism in the female reproductive tract. Mice deficient in *Loxl1* (KO) fail to polymerise tropoelastin monomers to generate elastin polymers through lack of a crosslinking mechanism mediated by the *Loxl1* enzyme. Studies using these KO mice provide mechanistic evidence for defects of elastin homeostasis in the ECM in the pathogenesis of POP. The LOXL1 (lysyl oxidase, LOX) enzyme is essential for the extensive remodeling of the female reproductive tract during pregnancy, birth, and following parturition. *Loxl1* KO mice develop a urogenital bulge and show weakened vaginal biomechanical properties [25]. They do not recover sufficiently from vaginal birth trauma which then progresses to POP in parous mice [82]. Fibulin-5 (FBLN5) is an elastin-binding protein that localizes to the surface of tropoelastin monomers to promote elastogenesis by monomer self-aggregation [27]. Mice deficient in *Fbln5* gradually develop a urogenital bulge as they age, mimicking the occult progression of POP seen in women years after vaginal birth [83]. There is also an influx of monocytes and macrophages into the vagina of *Fbln5* KO mice, which likely release proteolytic enzymes that attack the ECM and further weaken the vaginal wall. These KO models provide insights into POP pathophysiology and the important role of the ECM. While most women with POP do not have genetic mutation in the *Loxl1* gene, KO models also guide translational studies focused on deeper phenotyping of POP, which is essential for the development of novel therapeutics. For example, the study by Alarab et al. found that the expression of LOX, LOXL1, and LOXL3 genes and the corresponding proteins is significantly reduced in the anterior vagina of premenopausal women with advanced POP (*n* = 15) compared to that in the analogous specimens procured from age- and menstrual cycle phase-matched controls (*n* = 11) [84].

### POP Treatment Animal Models

A wide range of animal models have been used in evaluating novel nondegradable and degradable meshes and tissue engineering constructs for treating POP, ranging from mouse wound repair, rat vaginal distention models of POP, sheep vaginal surgery models to the use of macaques and dogs. Several excellent reviews highlighting the features, advantages, and disadvantages of these animal models are recommended [85, 86]. Outcome measures varied widely between studies, ranging from semi quantitative histology scoring [87] to quantitative image analysis of immunostained sections to quantify immune cell numbers, neuromuscular junctions [88], or measure gradients of response [44] around mesh filaments. Newly deposited collagen (type III) versus stable collagen (type I) has been assessed by quantitative Sirius Red fluorescence [77]. Other outcomes include M1 and M2 macrophage response, angiogenesis, and gene expression (reviewed in Verhorstert et al.) [89]. Uniaxial and multiaxial biomechanical evaluation of explanted vaginal mesh-tissue complexes is important and possible in the larger preclinical animal models [90]. Assays of vaginal smooth muscle function, such as vaginal contractility, receptor function, innervation, and innervation density on explanted mesh-tissue complexes provide functional data [91]. Nanoscopic methods such as atomic force microscopy and scanning electron microscopy have also been used to characterize explanted meshes [92]. One study selected parous sheep on the basis of objectively measured vaginal wall weakness and distributed the sheep within each experimental group to ensure similar levels of “POP” [93]. Cells included in the tissue engineering constructs need to be tracked in the harvested tissue. Genetic methods such as lentiviral labeling with mCherry can be used [94] or fluorescent supra-paramagnetic iron oxide nanoparticles (SPIONs) as exemplified when autologous ovine endometrial MSC were vaginally implanted with a novel polyamide/gelatin knitted mesh [45]. Some studies have compared vaginal versus abdominal mesh implantation [95]. Given the volume of new mesh biomaterials and hydrogels currently being developed and evaluated in animal models of POP, it is important that a standardized set of outcome measures are used. Outcome measures for assessing vaginally and non-vaginally implanted biomaterials and tissue engineering constructs have been suggested for preclinical models to enable comparison between studies [89]. The International Urogynecology Association (IUGA) has also recommended a standardized description of graft-containing meshes [96].

## Prevention-Focused Interventions

The lifespan model of pelvic floor (dys)function, described by DeLancey et al. in 2008, divides risk factors for PFD, including POP, into three broad phases: predisposing factors (phase I), inciting factors (phase II), and intervening factors (phase III) [97]. The knowledge of specific factors within each phase is necessary to develop effective primary, secondary, and tertiary preventative strategies for POP that can be personalized for different individuals. To this effect, we will focus on the major predisposing, inciting, and intervening risk factors for POP, identified by the epidemiological studies in patients with POP and perturbed in preclinical models.

### Primary POP Prevention

Deeper phenotyping–genotyping is necessary to screen and risk stratify patients before prolapse-inciting events occur. Identifying specific phenotypes that precede the onset of POP, a heterogeneous and multifactorial condition, is vital to develop primary prevention strategies.

#### Predisposing Factors: Genetics

Genetic predisposition to POP is suggested by clinical research studies that demonstrate a high concordance of prolapse in twins and an increased risk of prolapse among women with connective tissue disorders [98, 99]. Furthermore, a 2015 systematic review and meta-analysis found evidence supporting the association between a specific collagen gene variant (*rs1800012* in *COL1A1*) and POP. In preclinical studies, murine KO models have helped elucidate the contributions of specific genes coding for proteins important for elastin and collagen assembly and ECM homeostasis to POP development due to compromised recovery after parturition [37] or age-related impairment [100], as described above [27].

Despite evidence that POP is heritable [27], numerous studies aimed at identifying genetic loci associated with POP have identified only a small number of biologically plausible variants, and functional validation studies in humans have not been performed [101]. Using the Utah Population Database (UPDB), a unique database that links genealogy data to medical records for ~ 85% of the state of Utah, Allen-Brady et al. found that the risk of POP treatment increases with the number of relatives treated for POP, ranging from a relative risk of 2.4 (95% CI 2.2–2.6) with 1 first-degree relative up to 6.3 (95% CI 1.3–18.2) with 3 first-degree relatives [102]. Despite this association, POP predisposition genes remain elusive. A recent meta-analysis of genetic association studies for POP identified four polymorphisms in hormone receptor and ECM genes ESR1, FBLN5, PGR, and COL1A1 [27]. While these genes are biologically credible, the epidemiological reliability for each variant was moderate. Another recent meta-analysis of two genome-wide association studies (GWAS) evaluating over 14,000 patients with POP identified eight variants at seven loci that met genome-wide significance [103], though none were coding variants or in high-linkage disequilibrium with coding variants, complicating the interpretation of functional consequences of these variants. Nonetheless, even polymorphisms identified in noncoding regions may represent potential targets for genetic and molecular-targeted therapies.

A major limitation of genetic studies is the inability to control for phenotypic heterogeneity. To address this limitation, Pujol-Gualdo et al. developed a polygenic risk score (PRS) for POP using 3,242,959 single nucleotide polymorphisms (SNPs) in addition to clinical characteristics like asthma, BMI, constipation, parity, and smoking, which performed moderately well (C-stat = 0.630) in predicting incident POP [104]. Clinical applications of PRS are currently under investigation for cardiovascular disease, type 2 diabetes, breast cancer, and Alzheimer’s disease [105]. While studies are needed to validate the POP PRS and explore other potential phenotypic associations, the combination of genetics and clinical factors to develop a personalized risk score could be a highly valuable tool in risk stratification and predictive modeling. Improving our understanding of the genetic predisposition to POP is critical to our ability to establish a comprehensive disease model, develop early intervention strategies, and identify novel therapeutic targets for POP.

## Secondary and Tertiary POP Prevention

### Inciting Events

Childbirth (title of subsection) A large body of epidemiological literature identifies vaginal parity as the strongest risk factor for symptomatic POP. Despite this, mechanistic studies that causatively link maternal birth injury and POP are scarce.

#### Tissue Remodeling/Postpartum Recovery

Injury and impaired recovery of pelvic soft tissues are putative mechanistic links between vaginal delivery and subsequent POP. A critical component of pelvic floor support is the genital hiatus, or vaginal opening. A normal genital hiatus is established through interactions between the levator ani muscles, paravaginal connective tissue attachments, and resting tone of the vaginal smooth muscle. Impairment in any of these structures can cause genital hiatus enlargement. While an enlarged genital hiatus is associated with POP, it has only recently been shown to *precede* POP development. In a seminal longitudinal study of ~ 1200 parous women, Handa et al. discovered that genital hiatus enlargement remote from delivery [106] preceded symptomatic POP. A more recent longitudinal study of 580 primiparous women from the 3rd trimester to 1 year postpartum found that compared to those with a genital hiatus size < 4 cm at 8 weeks postpartum, women with a genital hiatus size of ≥ 4 cm had a 3.3-fold increased risk of POP at 1 year postpartum [107], suggesting that genital hiatus enlargement starts as early as the first postpartum year.

Genital hiatus enlargement is directly related to vaginal delivery as opposed to pregnancy-related changes. In a study of primiparous women in the first postpartum year, those who delivered vaginally had a 50% larger genital hiatus compared to those who had a cesarean delivery [108]. One possible explanation for this finding is levator ani avulsion at the time of vaginal delivery, which is associated with a nearly 50% increase in genital hiatus size and 7.3-fold increased odds of POP [109]. However, levator ani avulsion is only present in approximately half of POP cases, pointing to the likely existence of other injury phenotypes demonstrated in preclinical simulated birth injury models [110] or maladaptive recovery responses that account for the association between vaginal delivery, genital hiatus enlargement, and POP. For example, older maternal age at first vaginal delivery (≥ 33yo) significantly and independently increases both genital hiatus size and prevalence of POP at 1 year postpartum [111]. These findings suggest that vaginal delivery may lead to POP as a result of impaired recovery of pelvic soft tissues, augmented by an age-related decrease in endogenous regenerative capacity.

Impairments in pelvic floor ECM components, particularly collagen and elastin, have been associated with POP in animals and humans [112]. *LOXL1* KO mice demonstrate advanced POP 1 to 3 days postpartum after delivering their first or second litter [82]. While no genital hiatus measures were made, Liu et al. found that the vaginal circumference was several-fold larger in *LOXL1*^*−*^*/*^*−*^ compared to wild type mice. The decrease in the ultimate load at failure observed in the vagina–supportive tissue complex of the *LOXL1* KO mice indicates mechanically weaker tissues [25], which is a likely common endpoint of the multiple etiologies that progress to POP. Few human studies have looked at the role of LOXL1 in postpartum POP or pelvic floor recovery. Zhao et al. reported a ~ 60% reduction in expression of LOXL1 in the uterosacral ligaments of postmenopausal women with POP compared to controls [113]. Taken together, these preclinical data identify potentially fruitful therapeutic targets to prevent or mitigate POP. On the basis of this research, recent studies have examined the feasibility of novel hydrogels targeting ECM components like fibulin-5 to prevent ECM degradation and fibrosis. Good et al. developed and delivered a *fibulin-5 hydrogel* to the vaginal wall in mice that inhibited MMP-9 and prolonged the half-life of fibulin-5 in cultured fibroblasts in vivo [114]. Similar hydrogels could be developed for LOXL1 and other critical enzymes found to play a critical role in POP development.

Childbirth-induced pelvic floor muscle (PFM) injury is another well-established epidemiological risk factor for POP. The Alperin and Christman labs deployed a skeletal muscle ECM hydrogel derived from decellularized porcine skeletal muscle that mitigated PFM atrophy and fibrosis when administered either at the time of birth injury or 4 weeks post-injury in a preclinical rat model of simulated birth injury [54]. While neither of these studies involved humans, promising safety and efficacy data of decellularized porcine ECM hydrogels has been demonstrated in humans after myocardial infarction [115].

While most studies have demonstrated the association between vaginal delivery and PFM injury via macroscopic injuries visible on ultrasound or magnetic resonance imaging (MRI), microscopic injuries also likely occur that impair muscle function and predispose to POP development. Muscle stem cells (MuSC) are indispensable for skeletal muscle regeneration and, along with the supporting fibro-adipogenic progenitors (FAPs) and immune cells, proliferate after muscle injury. Using a validated birth injury rat model, Sesillo et al. demonstrated that MuSCs and FAPs proliferate as early as 3 days after birth injury, leading to constructive tissue regeneration [88]. In rats with impaired regeneration induced by PFM irradiation that precluded MuSCs and FAPs from replicating, the regenerative process does not start until 7 days post-injury and is less robust, leading to PFM atrophy long-term. These findings raise the possibility of utilizing MuSCs or FAPs as therapeutic targets to enhance PFM regeneration following vaginal delivery, particularly in women at high risk of pelvic floor injury [88].

### Intervening Factors

In addition to discrete inciting events like birth injury, epidemiological studies identify aging as the top *promoting risk factor* for symptomatic POP. Aging is the process of cellular senescence characterized by cell cycle arrest and inflammaging [116]. Other aging phenotypes that have been identified at the cellular level include genomic instability, telomere attrition, epigenetic alterations, loss of proteostasis, deregulated nutrient sensing and intercellular communication, mitochondrial dysfunction, and stem cell exhaustion [103]. Despite the indisputable association between aging and symptomatic POP development, the causative mechanisms linking aging and POP are yet to be elucidated.

#### Aging

POP may result from age-related cellular and molecular changes impairing pelvic soft tissue homeostasis, regeneration, and function. Various studies correlate SASP with POP, suggesting that cellular senescence may be an important pathophysiologic mechanism of POP development and exacerbation. Senescent cells negatively affect surrounding tissues because they are in a state of cell cycle arrest yet remain metabolically active, thus exhausting proliferative and renewable stem cells over time. Senescent fibroblasts interfere with the regenerative and homeostatic capacity of pelvic tissues by deactivating transforming growth factor-β (TGF-β) and stimulating pathways that arrest cell cycle progression. A recent study by Florian-Rodriguez et al. was the first to induce senescence in vaginal fibroblasts in rats using etoposide, a topoisomerase II inhibitor that induces senesnces through double-strand breaks in DNA [117, 118]. A dose-dependent increase in SASP pro-inflammatory factors in vaginal fibroblasts was observed with in vitro etoposide treatment. The senescent fibroblasts were treated with a combination of dasatinib and quercetin. Decreased SASP expression and lower cell count were observed, suggesting that these senolytics restore cellular apoptotic capacity, in turn, facilitating clearance of senescent fibroblasts in vitro. Individually, dasatinib is a kinase inhibitor and quercetin modulates transcription factors by binding to BCL-2, a protein that regulates apopotosis. Together, dasatinib and quercetin have been shown to work synergistically to clear senescent cells and decrease pro-inflammatory cytokines in numerous animal models as well as human adipose tissues [119, 120]. A more recent study from this group demonstrated the ability to quantify SASP markers from vaginal secretions using multi-plex immunoassays of vaginal swabs in 81 women (4 groups: pre- and postmenopausal women, with and without prolapse), with the highest concentration of SASP markers in postmenopausal women with prolapse and the lowest in premenopausal women without prolapse [121]. Stem cells may also modulate the effects of senescence-induced pelvic floor changes. In a study by Wen et al., induced pluripotent stem cells (iPSC) were successfully isolated from vaginal fibroblasts of a 47 yo and a 78 yo women [122]. The stem cells were re-differentiated to fibroblasts to compare the SASP marker senescence-associated galactosidase activity and mitotic index, which did not differ between the two women. The authors concluded that older age does not interfere with successful reprogramming of stem cells isolated from vaginal fibroblasts. In another in vitro study by Li et al., umbilical cord-derived stem cells attenuated the cytotoxic effects of SASP markers on fibroblasts from women with POP by inducing anti-inflammatory pathways [123]. These studies suggest that cellular senescence and SASP-associated inflammaging play a role in POP development and that senolytics, such as dasatinib, quercetin, and fisetin, a flavonoid present in many fruits and vegetables that is currently being tested in a phase 2 clinical trial as described above, have potential as a novel preventative and/or therapeutic strategy for POP.

#### Menopause

Menopause is characterized by hypoestrogenism that impacts pelvic soft tissues through a myriad of hormonally mediated, tissue-specific changes. Critical effects of hypoestrogenemia on tissue integrity include a decrease in collagen I relative to collagen III [124, 125], as well as increased expression of matrix metalloproteinases [126, 127] that actively degrade collagen, which together lead to a deterioration in the biomechanical properties of the vagina and its associated supportive tissues. This association was illustrated in a study by Moalli et al. [128] in which the effects of ovariectomy (OVX) on two key biomechanical features of the vagina, load-to-failure and linear stiffness, were evaluated in a rat model. The study found that OVX (i.e., surgical menopause) in 4-month Long-Evans rats yielded a 40% decrease in linear stiffness and a 30% decrease in load-to-failure relative to sham-operated controls, consistent with more distensible and weaker vaginal tissue. Estrogen supplementation after OVX in these 4-month rats mitigated the decreases in linear stiffness and load-to-failure, supporting hypoestrogenemia as the cause of pelvic tissue biomechanical decline. Biochemical studies also illustrate the negative effects of hypoestrogenism on pelvic tissue integrity. In a study by Rizk et al. [129], the impact of hypoestrogenism on p27kip1, a cyclin-dependent kinase inhibitor responsible for striated muscle differentiation and apoptosis, was evaluated in 13-month-old OVX Wistar rats. The study demonstrated a significant increase in cytoplasmic signal intensity of p27kip1, consistent with striated muscle cellular aging, after OVX compared to sham-operated animals in striated urethral sphincter, striated anal sphincter, and levator ani muscle. OVX-caused hypoestrogenemia was associated with increased MMP expression, decreased collagen I:collagen III ratio, and striated muscle cellular senescence, presumed to govern deterioration of vaginal supportive tissue complex biomechanical properties.

#### Hormonal Therapy

Because of the increased rates of POP seen in postmenopausal women and the known impacts of hypoestrogenemia on pelvic floor tissue biochemical and biomechanical properties, local and systemic hormonal therapy for POP prevention has been extensively evaluated. Despite decades of research, the effect of hormonal therapy on POP outcomes in postmenopausal women remains controversial, and studies have reported conflicting results. A case–control study reported that menopause hormone therapy (MHT) use for at least 5 years after menopause was associated with a lower rate of PFDs [130]. Another study revealed that MHT in postmenopausal women did not decrease the overall risk of POP development but was associated with reduced severity of POP [131]. While other studies have found the impact of MHT on pelvic organ support to be minor and of low clinical relevance [132], these were retrospective observational studies that complicates interpretation of results.

From a mechanistic standpoint, Wang et al. studied the effects of 17β-estradiol (E2) on pelvic fibroblast proliferation, apoptosis, and protein expression at different concentrations and time intervals. Increasing E2 concentrations were added to cultures of primary uterosacral ligament fibroblasts, and the authors reported both concentration- and time-dependent effects of E2 on cell proliferation, as well as pro-collagen and estrogen receptor expression [133]. Other studies have evaluated selective estrogen receptor modulators (SERMs) as preventive therapies for PFDs. Goldstein et al. assessed the effects of raloxifene on the frequency of surgery for prolapse in postmenopausal women and found that raloxifene was associated with a reduced risk for pelvic floor surgery [134]. A subsequent study by Lee et al. sought to identify potential mechanisms by which raloxifene may mitigate POP development and found that raloxifene selectively attenuates abnormal matrix degradation by increasing protease inhibitors (TIMP-3, TIMP-1, and TIMP-3) in pelvic fibroblasts [135]. Contrary to findings from these studies, preclinical evaluation of the effects of five different SERMs on the function and ECM components of the vagina and its supportive tissues in rats found that SERM supplementation did not prevent deterioration of the biomechanical properties of the vagina and its supportive tissues associated with estrogen deprivation, with idoxifene and bazedoxifene being the least effective. The investigators observed a paradoxically increased collagen content in idoxifene- and bazedoxifene-treated groups, likely related to increased formation of nonfunctional collagen [136]. Importantly, a recent robust superiority RCT by Rahn et al. demonstrated that adjunctive perioperative vaginal estrogen did not improve surgical success rates up to 36 months after native tissue transvaginal prolapse repair [137], further complicating our understanding of the impact of estrogen on POP development, progression, and recurrence.

#### Obesity

Obesity has been identified as a modifiable risk factor for POP and other PFDs. This relationship is thought to be due to the increased intra-abdominal pressure exerted by excess weight, which can cause structural damage or neurological dysfunction to the pelvic floor [138]. Several studies have shown that women who are overweight or obese (body mass index [BMI] > 30 kg/m^2^) have up to a 2.5-fold increase in risk of POP and other PFDs when compared with women of normal weight [139–141]. Weight loss, whether through diet or bariatric surgery, has been suggested as a preventive measure for PFDs. While weight loss has been associated with subjective improvement in prolapse symptoms, the impact on objective metrics obtained using the pelvic organ prolapse quantification (POP-Q) system is less clear [142]. Cuicchi et al. found that the prevalence of pelvic floor dysfunction decreased with weight loss after bariatric surgery, resulting in a significant improvement in quality of life [143]. Gozukara et al. found that weight reduction resulted in significant changes in pelvic floor anatomy, but the nature of these changes and their impact on improvement of urinary incontinence and pelvic floor-related symptoms are uncertain [144]. Pomian et al. found that bariatric surgery is associated with a betterment of bladder neck position at rest, with pelvic floor muscle contraction, and with strain in women without PFDs. This suggests that bariatric surgery may be used as a tool for PFD prevention, but it does not improve levator ani function or limit bladder neck hypermobility, implying that it has no influence on preexisting pelvic dysfunction. In addition, weight loss does not appear to restore muscle function or repair existing damage to the pelvic floor. Consequently, while weight loss may help prevent the development or progression of PFDs, it is unlikely to be sufficient to reverse existing POP [145].

### Current Tools to Help Guide Preventative Strategies

#### Imaging

Imaging studies have played a pivotal role in advancing our understanding of POP and guiding primary, secondary, and tertiary prevention strategies. Using MRI and ultrasound, dynamic changes in pelvic anatomy and structural alterations in pelvic supportive structures associated with POP have been identified. Known predictors of POP development and postoperative POP recurrence identified by advanced imaging techniques include enlarged genital hiatus size [146], failed hiatal closure [147], paravaginal tissue location and integrity [148], inferior descent of the vaginal apex and perineal body [149], and angulation of the perineal body [150]. Weaker pelvic floor muscles have also been identified as an independent risk factor for POP development [151]. Recently, Pipitone et al. developed and validated a novel MRI-based perineal membrane reconstruction technique to determine the effects of pregnancy and childbirth on the perineal body. They found that women after VD had 13% larger separation at the perineal body compared to nulliparous women (*p* = 0.097, Cohen’s *d* = 0.84) and 23% larger hiatal area compared to women who underwent cesarean delivery (*p* = 0.14, Cohen’s *d* = 0.94), concluding that perineal membrane morphology and perineal body separation after VD may predict future POP development [150].

Levator trauma is a particularly important risk factor for POP. Ultrasound and MRI studies indicate that up to 30% of women after VD demonstrate levator avulsion injury [152, 153]. Handa et al. reported that women with levator avulsion had a significantly higher rate of prolapse than those without avulsion (OR 4.17, 95% CI 2.28–7.31), and risk of POP was positively associated with levator hiatus area (OR 1.52 per 5 cm^2^, 95% CI 1.34–1.73) [151]. While levator avulsion is associated with abnormal hiatal area with strain and decreased pelvic floor muscle strength, data suggest that even without macroscopic levator trauma, there may be increased hiatal distensibility that leads to future hiatal enlargement and POP. In a prospective study, Shek and Dietz showed that VD with levator avulsion was associated with a 28% increase in hiatal area, while delivery without levator avulsion increased hiatal area by 6% [154]. Failure of the levator and urogenital hiatuses to remain closed is strongly associated with PFDs [147], and women with enlarged urogenital hiatus (≥ 3.5 cm vs ≤ 2.5 cm at Valsalva) have prolapse of 11.7 (95% CI 7.51–18.4) [155]. Another study by Barca et al. demonstrated a significant correlation between age over 35 years in pregnant women and increased genital hiatus area (*r* = 0.295, *p* = 0.031) via 3D ultrasound. Using this noninvasive and reproducible imaging technique at 20 weeks of gestation, the authors suggest that labor prognosis and potential for future perineal dysfunction can be predicted, providing a window of opportunity to implement preventive measures that reduce the likelihood of POP development [156].

Preventing prolapse recurrence after surgical repair is another concern, with studies reporting failure rates up to 36% at 2 years [157]. In a retrospective cohort study by Wyman et al., a novel MRI assay of estimated levator ani subtended volume (eLASV) was evaluated and shown to predict surgical failure after laparoscopic bilateral uterosacral suspension when exceeding a calculated optimal cutoff point of 38.5 cm^3^ [158]. Understanding individual risk factors and leveraging novel imaging assays like eLASV for early detection of POP-associated biomarkers will enable pelvic floor specialists to tailor preventive measures and provide targeted advice to patients. Furthermore, regular imaging assessments using noninvasive and reproducible techniques may identify subtle changes in pelvic organ position that permit timely adjustments to treatment plans and preventive strategies. Integrating imaging into pelvic floor health may enhance our ability to address both primary risk factors and early signs of prolapse, ultimately contributing to more effective prevention and management strategies.

#### Genomic and Proteomic Testing

A key factor in identifying POP prevention strategies is improving our mechanistic understanding of POP etiology. Researchers can identify potentially critical differences in genes, proteins, and cells associated with POP utilizing bioinformatics. While the GWAS studies described above produced conflicting results, Cox et al. identified various SNPs (*rs12325192, rs93068s94, rs1920568,* and *rs1247943*) that may contribute to POP susceptibility using a database linking genetic and clinical data from patients with POP [159]. Wang et al. screened differentially expressed genes (DEGs) in POP (*n* = 32) and control patients (*n* = 34) and a total of 10 hub genes were identified, namely *cdkn1a, il-6, pparg, adamts4, adipoq, areg, atf3, ccl2, cd36*, and *cidea*, that might be closely related to the occurrence of POP. Also, they found a higher abundance of genes related to CD8^+^ T cells in three datasets (GSE53868, GSE28660, and GSE12852) that negatively correlated with follicular helper T cells or gamma T delta cells and positively correlated with M2 macrophages and memory CD4 T cells in patients with POP [160]. Other studies have identified DEGs in pelvic soft tissues from women with vs without POP that are related to ECM structure and function, metabolic regulation, and immune function [161–164]. In particular, DEGs were observed in adaptive immune response, T cell differentiation, and T cell activation [164]; greater infiltration of activated mast cells and neutrophils into POP vs non-POP tissues [162]; and negative regulation of growth and inflammatory responsiveness [161]. Zhou Q et al. [165] utilized microarray data for GSE53868, which included 12 anterior vaginal wall samples from women with and without POP. Upregulated DEGs in the POP group were predominantly associated with immune response, complement activation, classical pathway, phagocytosis, and recognition. Meanwhile, downregulated genes were largely linked to cellular response to zinc ion, negative regulation of growth, and the apoptotic process. The protein–protein interaction (PPI) network helped identify *il6, myc, ccl2, icam1, ptgs2, serpine1, atf3, cdkn1a*, and *cdkn2a* as key genes in the development and progression of POP. For instance, negatively regulated IL-6 has been associated with PFM weakness observed in women with POP. IL6 plays a crucial role in muscle growth and repair; thus, suppression of IL-6 reduces muscle anabolism, leading to decreased muscle mass and strength. Furthermore, reduction in IL-6, which is an important component of the inflammatory response necessary for muscle repair after exercise or injury, could impair muscle recovery [166, 167]. Other factors such as cell proliferation and apoptosis, influenced by the *myc* gene, also play a role in POP development. The protein Myc works against cell cycle inhibitors like p21 and p27 through various mechanisms [168]. While research on cell cycle regulatory proteins in relation to POP is limited, Yamamoto et al. [169] were the first to suggest a connection between cell cycle regulation, cell proliferation, and POP development. Their research showed enhanced proliferation abilities in fibroblasts from human cardinal ligaments, along with significantly reduced levels of p53 and p21 (proteins that typically cause G1 phase arrest) in POP patients compared to controls. This suggests that when p53 and p21 are deficient, fibroblasts in the pelvic support system cannot properly enter their resting state. This leads to increased fibroblast proliferation, which consequently results in decreased production of elastin and other extracellular matrix components that are normally produced during the cell’s quiescent phase [170]. CCL2, functioning as an immunoregulatory and inflammatory cytokine, along with the ICAM1 protein node, which acts as a wound healing regulator, are involved in ECM repair within pelvic floor tissue and muscle following injuries sustained during pregnancy and childbirth [171]. *PTGS2, CDKN1A*, and *CDKN2A* are also associated with POP progression, affecting prostaglandin biosynthesis, cell proliferation, apoptosis, and DNA damage repair. Prostaglandins modulate peripheral microvascular reactivity and distribution which play a crucial role in maintaining the supportive tissues [172]. The roles of *SERPINE*1 and *ATF3* in POP development and progression require further investigation. In a 2020 study, Zhao et al. [162] used microarray data from 34 samples procured from 16 participants with POP and 18 without. A differential gene expression analysis was validated with RT-qPCR. Functional annotation showed that DEG were primarily involved in immunity. The identified hub genes, *znf331, thbs1, ifrd1, flj20533, cxcr4, gem, sod2*, and *sat*, were overexpressed in POP compared to non-POP tissues. The hub genes were involved in regulating immune-related pathways, such as the IL-17 signaling pathway, which has been linked to immunopathology and autoimmune diseases. Authors speculate that the IL-7 signaling pathway plays a significant role in POP development due to its role in immune regulation as the immune system plays a role in maintaining the homeostasis of the pelvic tissue extracellular matrix. In their study, Yu et al. analyzed 12 samples of uterosacral ligaments (USLs) from 6 women with and 6 without POP through transcriptomic and metabolomic analyses [164]. They identified 487 DEGs between the POP and control groups. A functional enrichment analysis revealed these genes were again predominantly related to immune responses, such as adaptive immune response, T cell differentiation, and T cell activation. Key nodes in the PPI network included *PTPRC, LCK, CD247, IL2RB, CD2, CXR5, JUN, CD3E, IL2RG*, and *PRF1*. The integrated analysis showed that the DEGs included *nt5c1a, gmpr, sds, alas2, carns1, pycr1, p4ha3, pgs1*, and *nmrk2*. These genes encode enzymes involved in various metabolic pathways. Two DEGs, *sds* and *p4ha3*, are directly involved in collagen metabolism. *sds* encodes one of three enzymes critical in serine and glycine metabolism, while *p4ha3* encodes a key enzyme in collagen synthesis, known to promote cellular proliferation, migration, and clonogenicity. Even though, these DEGs have not previously been reported in the molecular profile of POP, authors suggest that they may be used as new therapeutic targets, following further experimentation for validation. Li et al. used single-cell RNA-seq to construct a transcriptomic cell atlas of the vaginal wall in anterior prolapse by using isolated vaginal wall cells from the prolapsed anterior vaginal wall of 16 patients and the anterior vaginal wall of five control individuals. They found that intercellular communication was altered in POP tissues with increased interactions between smooth muscle cells, fibroblasts, and macrophages in POP samples vs controls [173]. The authors propose that altered cell interactions may disrupt normal cellular function, contributing to POP development. Notably, the study found that immune cell dysfunction was present in the POP tissues. The authors hypothesize that this immune cell dysregulation leads to disordered interactions with the ECM during tissue injury which could impair tissue repair. Furthermore, tissue remodeling-related interactions, such as the *TGFB1–TGFBR2* interaction, were gained in smooth muscle cells, indicating these cells’ participation in the ECM remodeling. The authors suggest that the phenotypic switch from smooth muscle cells to myofibroblasts in the vaginal wall could be the underlying cause of structural changes in the muscularis seen with POP in which increased deposition of collagen type III occurs [174, 175], resulting in thinner and more fragile vaginal tissues.

#### Risk Predictive Models

Various models have been developed to predict the risk of POP after childbirth or of recurrence after POP treatment. Jelovsek et al. developed and validated a prognostic model using ante- and intrapartum variables to estimate the risk of PFDs 12 and 20 years postdelivery [176]. Not surprisingly, the model indicated that route of delivery and family history of PFDs were strong predictors of POP. Another prognostic model to predict the risk of anatomical anterior prolapse recurrence, after surgery for POP, used translabial 3D ultrasonography together with various clinical variables, including history of assisted delivery, preoperative POP stage, presence of levator defects, and levator hiatus size [177]. Additionally, Zhang et al. established a multidimensional predictive model for postoperative recurrence of POP by constructing a nomogram that incorporated age, BMI, preoperative stage of prolapse, preoperative GH, and serum calcium to predict recurrence 1–2 years after POP surgery. The constructed nomogram model had suitable identification ability (AUC = 0.9) which refers to the model’s power to distinguish between different classes or outcomes correctly and ranges from 0.5 to 1.0 (the closer the AUC value is to 1, the better the identification ability of the prediction model), calibration (c2 = 29.3, *p* = 0.52), and clinical applicability indicated by the alignment between expected recurrence rate and actual recurrence rate [178].

## Advancements in Treatment-Focused Interventions

### Personalized Devices

While vaginal pessaries have been used as a nonsurgical treatment option for POP since ancient times, up to 30% of patients with POP cannot be successfully fitted with standard pessaries. Personalized, patient-specific pessaries fabricated using 3D printing may be a potential solution for these patients and those imperfectly fitted with mass-produced pessaries. Despite several recent case reports on 3D-printed pelvic floor devices, urogynecology is trailing behind other fields in developing and using customized prostheses to improve patient care. One pilot feasibility study helped to bridge this gap by assessing the use of 3D-printed, patient-specific pessaries to improve POP-related symptoms and increase overall satisfaction with pessary use in patients who have been previously unsuccessfully fitted with standard pessaries. The results demonstrated an improvement based on the Pelvic Organ Prolapse Distress Inventory 6 questionnaire and overall satisfaction on a visual analog scale after patient-specific pessary use, demonstrating the feasibility of pessary personalization to improve current conservative treatments [179]. By leveraging 3D printing technology together with rapid advancements in artificial intelligence, there is potential for a paradigm shift in the design of vaginal pessaries based on patient-specific anatomy [180]. AI can be leveraged to obtain patient-specific anatomy from standard scans, which can be used to produce precise pessaries for patients using 3D printing.

Although the above studies are limited by a small sample size, the rapid development of new technologies and their application to various fields of medicine indicate that in the near future we will witness a revolution toward personalizing the management of POP. In addition to individualizing pessary shape and size, it will also be possible to use pessaries for intravaginal drug delivery. For example, a research group in Auckland, New Zealand had recently developed an estradiol-eluting pessary. This device has mechanical properties in line with commercially available pessaries, while also releasing a constant dose of estriol over a period of 3 months, similar to the estrogen ring. This estradiol eluting pessary has the potential of becoming the first line treatment for POP in menopausal women [181].

### Radiofrequency

Pelvic floor therapy with electrical stimulation has been used as a nonsurgical intervention for bothersome “vaginal laxity” and stress urinary incontinence (SUI). Dyspareunia resulting from surgical introital narrowing is often a worrisome complication. A noninvasive method for altering tissue compliance in the vaginal introitus is nonablative radiofrequency (RF) energy. SUI has been treated with transurethral monopolar radiofrequency-induced collagen denaturation, which carries a low risk of side effects. On the basis of thermal tissue remodeling as opposed to ablation, radiofrequency radiation has also demonstrated a significant and safe track record of noninvasively treating rhytides (wrinkles) in the delicate tissue of the periorbital area and the loose skin of the face and neck [182].

RF is a potential minimally invasive treatment option for POP. In the nineteenth century, Arséne D'Arsonval’s stipulated that the passage of high frequency electric current through the tissues of a living person could have a potential therapeutic effect. The emission of high-frequency electromagnetic waves produces heat within treated tissues, increasing tissue metabolism. At present, RF is considered a type of wave or electromagnetic radiation with high frequency that is in the category of nonionizing radiation and whose use is widespread in medicine and kinesiology. Irreversible cellular damage in biological tissues occurs at 50–55 °C. The “nonablative” RF used in kinesiology is administered by means of short high-voltage pulses, which can reach temperatures of around 40–42° C, with heat dissipation between pulses, preventing tissue coagulation. The “ablative” RF used to treat pain or cancer and for electrocautery is administered continuously without periods in which the heat is dissipated, with temperatures reaching 80–82 °C.

With respect to pelvic soft tissues, RF has been shown to stimulate proliferation of glycogen-enriched epithelium, collagen, and neovascularization, when applied to the vagina. At temperatures between 40 °C and 45 °C, an inflammatory cascade is initiated, with activation of heat shock proteins that stimulate fibroblast proliferation and differentiation, in turn, leading to increased production of fibrillar ECM components [183]. The above notion is supported by the findings in preclinical models. In 2018, Kent et al. examined the effect of RF delivered via intravaginal applicator on vaginal collagen and elastin content in domestic pigs (*n* = 3) [184]. Animals were treated once a week for 3 weeks. Punch biopsies were obtained from mid- and proximal portions of the vagina for histological evaluation immediately following each treatment and at 1 and 4 weeks following the last treatment. Specimens were obtained via punch biopsy under ultrasound guidance. Histological assessments revealed a significant increase in elastin and collagen fiber density after each treatment, with the highest amounts observed at 1-week following the last treatment. The study also documented a higher nuclear density in H&E-stained vaginal sections obtained immediately following each treatment, presumed to be due to the increase in the number of fibroblasts. However, greater nuclear density could be due to inflammatory infiltrate, as the number decreased as early as 1 week following the last treatment. Furthermore, RF did not significantly increase the overall vaginal wall thickness assessed by ultrasound. The study concluded that RF treatment resulted in neocollagenesis and neoelastinogenesis in the porcine model, suggesting potential benefits for vaginal tissue remodeling [184].

Data on the role of RF in the treatment of POP are scarse. In 2021, Eftekhar et al. reported outcomes in 22 patients with “vaginal laxity” who underwent RF [185]. Women underwent pelvic organ prolapse quantification (POP-Q) examination and responded to the Female Sexual Function Index (FSFI-19) at baseline and 3 months after the intervention. This observational study found RF to be safe and to improve the objective and subjective outcomes. In a study from 2021 by Ghanbari et al., 43 patients with PFDs were treated with RF for three sessions [186]. Compared to the baseline, the investigators observed significant improvement in PFM function and subjective outcomes assessed by Female Sexual Function Index and Pelvic Floor Distress Inventory questionnaires 3-month post-intervention. Fu et al. treated 102 women with vaginal laxity with dual mode RF (monopolar and bipolar) [187]. Participants’ subjective outcomes, assessed with Vaginal Laxity Questionnaire (VLQ), Female Sexual Function Index (FSFI) questionnaire, and Sexual Satisfaction Questionnaire (SSQ), were compared pre- and post-treatment. Similarly to the other observational studies, RF was associated with improvement of vaginal laxity, sexual function, and PFM contraction. In addition to these small observational studies, level 1 evidence supporting the use of RF for treatment of vaginal laxity and the associated sexual dysfunction was provided by a sham controlled RCT conducted in 2017 by Krychman et al. funded by Viveve Medical, Inc. The primary endpoint, assessed using VLQ, revealed significant improvement in the active compared to the sham treatment group at the 6-month follow-up. Secondary outcomes, including the FSFI and revised Female Sexual Distress Scale (FSDS-R), were also improved in the active treatment group. The study concluded that a single session of RF therapy is a safe and effective nonsurgical approach for addressing vaginal laxity and improving sexual function [188].

The use of RF as treatment for PFDs has grown in recent years. In 2022, González-Gutiérrez et al. performed a a systematic review of 15 studies: 5 RCT and 10 other study designs (7 case series, 2 one-arm non-RCT and 1 prospective study). The authors concluded that RF could be effective for improving urinary incontinence, pelvic floor muscles’ strength, sexual function, and different pelvic pain conditions [183]. However, the overall existent evidence was deemed low-quality and none of the studies specifically targeted women with symptomatic POP.

### Vaginal Laser Treatment

Laser therapy is being increasingly deployed for the treatment of PFDs. Various types of lasers, including erbium and CO_2_ lasers, have been utilized for the treatment of vaginal laxity, sexual dysfunction associated with genitourinary syndrome of menopause and urinary incontinence. The putative mechanism by which lasers improve these conditions and possibly POP involves photothermal effects that stimulate collagen remodeling and neocollagenesis.

Fractional CO_2_ laser technology has been widely used in plastic surgery and dermatology with safe and effective outcomes afforded by the thermal effects of the CO_2_ laser on collagen remodeling. To assess the effects of fractional CO_2_ on vaginal tissues, Kwon et al. evaluated three levels of laser energy (60, 90, and 120 mJ) in a porcine model. Histological examinations of vaginal tissue demonstrated a dose-dependent increase in denatured lamina propria, showing that the impact of fractional CO_2_ laser was directly related to the amount of energy applied. Increase in vaginal collagen, specifically collagen I, and elastin, as well as angiogenesis were demonstrated in all groups. The laser treatment increased heat shock protein (HSP) 70 known for its role in protein folding and protection against thermal effects of laser induction, suggesting a positive effect on the tissue. These changes corresponded to the decrease in vaginal caliber, evident from a smaller internal diameter [189].

While a multitude of retrospective and prospective observational studies demonstrate efficacy of vaginal laser treatment for reducing symptoms of vaginal laxity and genitourinary syndrome of menopause (GSM) as well as improving sexual function, these findings have not been corroborated by RCTs, which have failed to demonstrate any or sustained differences in outcomes between laser vs sham treatments [190–193]. Further details regarding the conflicting results in the published studies can be found in the recent systematic review with meta-analysis [194].

Much less is known about the effect of laser treatment in women with POP. Sipos et al. conducted a prospective cohort study of 40 postmenopausal women with genitourinary symptoms of menopause who underwent three vaginal fractional CO_2_ laser treatments with 360° probe 4 weeks apart. Participants filled out the Pelvic Floor Distress Inventory (PFDI-20) questionnaire, which has three components: Pelvic Organ Prolapse Distress Inventory 6 (POPDI-6), Colorectal–Anal Distress Inventory 8 (CRADI-8), and Urinary Distress Inventory (UDI-6). POPDI-6 standardized scores showed no significant difference in prolapse symptoms after the first treatment. However, after the second treatment there was a significant improvement. UDI-6 standardized scores describe severity of the urinary distress symptoms. These were not significantly different after the first laser treatment. However, after the second and third treatments, study participants had significant improvement. CRADI-8 standardized scores characterize the colorectal–anal complaints of the participants. These values did not change significantly after three laser treatments [195]. Athansiou et al., in 2020, assessed the effectiveness of the non-ablative photothermal erbium laser (Er:YAG laser) as a treatment for anterior or posterior vaginal prolapse in a randomized single-blinded 1:1 trial comparing 3 Er:YAG laser (Intimalase Fotona SMOOTH™, Ljubljana, Slovenia) treatments administered for 3 months at monthly intervals to observation in postmenopausal women with symptomatic stage 2 or 3 prolapse (*N* = 15/group). The primary outcome was the proportion of participants with Stage 0 or 1 following laser treatment, while secondary outcomes included PFDI-20, Pelvic Floor Impact Questionnaire short-form (PFIQ-7), and Patients Global Impression of Improvement (PGI-I). All outcomes were evaluated at baseline and 1 month after the final treatment or after 4 months of observation. None of the participants in either group had POP-Q stage 0 or 1 at the study completion. Moreover, none of the participants in either group demonstrated any improvement in subjective outcomes [196].

### Microfocused Ultrasound Therapy

Microfocused ultrasound (MFU) is noted for its ability to penetrate deeper into the vaginal tissue, affecting the entire thickness without adverse effects on adjacent organs. MFU action relies solely on thermal effects, leading to collagen contraction and neocollagenesis [197]. Koloczewski et al. examined high-intensity focused ultrasound (HIFU) for the treatment of “vaginal laxity” and GSM. The outcomes include improvements in female sexual function, and vaginal health index. The study provided evidence of the effectiveness of MFU therapy in improving vaginal laxity, sexual function, and vaginal health in women with “vaginal laxity” and GSM [197]. MFU has not been deployed for the treatment of POP and the existing published studies lack control groups; however, deficiency in vaginal collagen content as well as altered proportions of various collagen isoforms have been demonstrated in women with compared to without POP.

### Novel Grafts for POP Reconstruction

The best graft materials replicate mechanical properties of healthy host tissues, do not cause significant complications, are easy to handle, and are not associated with exuberant costs. Unfortunately, synthetic polypropylene meshes are associated with mesh-related complications, such as mesh exposure, dyspareunia, and chronic pelvic pain, when placed vaginally. Moreover, these stiff materials can cause further atrophic deterioration of vaginal tissue by stress-shielding the host tissue. The above serves as an important impetus for modification of the existing materials and development of novel synthetic grafts for POP reconstruction.

To reduce complications associated with the overall mesh burden, Natalia et al. proposed “The Pelvic Harness”: a skeletonized mesh implant for safe pelvic floor reconstruction. The vaginal mesh implant was “skeletonized,” i.e., modified by removing about 75% of the total material, mimicking the ligamentary vaginal attachments. Ninety-five women with advanced POP were selected to undergo POP repair using the skeletonized scaffold in the form of a “ligamentary pelvic harness”. The postoperative anatomical outcomes were similar to those achieved with unmodified transvaginal mesh. In addition, the skeletonization did not increase surgical complexity compared to the unaltered mesh implant [198].

Another important factor thought to contribute to mesh-related complications is localized increase in the mesh burden and contraction due to collapse of mesh pores under predominantly unidirectional tension on vaginal meshes. To circumvent pore collapse, Knight et al. explored the behavior of auxetic meshes in silico [199]. In contrast to meshes with standard geometries, the auxetic materials have a negative Poisson’s ratio, i.e., they expand laterally under tension, effectively preventing pore collapse. Auxetic prosthesis have been used in cardiology and orthopedics and hold promise to significantly decrease the likelihood of mesh-related complications when deployed for POP reconstruction.

Motivated by the unacceptable rate of complications associated with polypropylene meshes, biologically active polymers have attracted significant attention for the development of novel natural or semi-synthetic materials due to their biocompatibility and widespread availability. Various scaffolds can be produced from a multitude of materials using electrospinning—a technique that uses an electric potential to create micro- to nanoscale polymeric fibers. Electrospun scaffolds have an adaptable large surface-to-volume ratio and a high porosity that promote cellular interactions, proliferation, and matrix deposition [200]. In addition, electrospinning is conducive to making implants that can also deliver various drugs or bioactive molecules. One such material, degradable melt electrospinning (MES)/melt electrowriting (MEW) construct [196], was 3D printed from a biodegradable polymer, polycaprolactone (PCL), and tested in the ovine model [201, 202]. PCL actively interacted with host tissue to promote healing and integration prior to complete degradation over a 2-year period. These new generation biomaterials promote endogenous cellular response resulting in tissue regeneration while providing a temporary mechanical support. The Guler group developed novel biodegradable estradiol (E2)-releasing electrospun poly-4-hydroxybutyrate (P4HB) scaffolds and tested its interactions with vaginal fibroblasts in vitro, with knitted P4HB serving as a comparator [197]. Owing to the differential microstructure, the electrospun P4HB induced increased cell proliferation and collagen and elastin deposition compared to knitted P4HB. However, E2 did not significantly impact vaginal fibroblast response. These novel materials are promising strategies for improving patient outcomes pending outcomes of the in vivo studies evaluating the host response and effects of mechanical loading.

## Safe Incorporation of New Treatments into Clinical Practice: Bench-to-Bedside-to-Market

While the main goal of this chapter is to outline innovations past, present, and those coming down the pipeline, we would be remiss not to discuss *safe practices* from preclinical investigations to translation and wide adoption into clinical practice. From discovery to preclinical investigation, to first-in-human, safety should be rigorously evaluated and reported. The process involves engagement with respective regulatory bodies, depending on the country of residence of the investigative team. This section will outline best practices based on expert consensus, and, when available, regional, national, and international guidelines.

### Preclinical Research

A crucial step in preclinical investigation involves selection of a model. The scope (cellular, tissue sample, organoid, animal), size, and duration of study with the model will largely depend on the research question. Ideally, models should be both tissue-relevant and site-specific. For the case of POP, that means that the vagina, supportive connective tissues, and pelvic musculature of the model are similar to human histologically (tissue-relevant), and the study involves use of the test article in the relevant pelvic structure of the model (site-specific). There may be instances where a test article is evaluated in a non-site-specific tissue prior to the site-specific one. For example, implanting sacrocolpopexy mesh in the abdominal wall to evaluate host response to the materials, and later implanting the same mesh in the vagina to evaluate biomechanical factors. The above considerations are important when designing experiments as these can impact study costs and might require modifications to satisfy local animal use guidelines, such as the Institutional Animal Care and Use Committee guidelines.

If a research question is on the molecular or tissue level, researchers may choose to use an in vitro model. Benefits of in vitro and ex vivo models include responsible animal use principles, high throughput, lower cost than in vivo experiments, and tighter control of the physical and chemical environment. Table 1 presents a non-exhaustive list of in vitro and ex vivo models that may be used in POP research. In vivo models are often used when the scope of the research question involves inter-play of local tissues or organs, if on-target and off-target safety studies are needed, and as a next-step after in vitro study. Table 2 presents common in vivo, or animal, models used in pelvic organ prolapse research. Several review papers are available on this topic [203–206].

**Table 1:** In vitro/ex vivo models available for use in pelvic organ prolapse research

Model type	Examples
Organoids Cell culture Bioactive hydrogels containing cellular or acellular therapeutics 3D Bioprinting Cellular and decellularized scaffolds Ex vivo biomechanical testing	Chumduri et al. [207] Alzamil et al. [208] Kang et al. [209] Feola et al. [210] Bachtiar et al. [211] Eberhart et al. [212] Florian-Rodriguez et al. [118] Wang et al. [133]

**Table 2:** In vivo models used in pelvic organ prolapse research

Species	Benefits of the model	Down-sides to the model	References
Rodents	Inexpensive Gross pelvic connective tissue is similar to humans	Pelvic muscles differ (e.g. levator muscles support tail function) Small fetus to pelvis ratio Small size Quadruped	Moalli et al. [125] Iwanaga et al. [213] Alperin et al. [25] Rahn et al. [214] Lee et al. [215]
Rabbit	Relatively inexpensive Larger than rodents and external vaginal is more accessible	Different vaginal anatomy – upper and lower vagina Upper vagina that connects to the uterus lacks adjacent supportive connective tissue	Alperin et al. [216] Huffaker et al. [217] Pero et al. [218] Knight et al. [219, 220]
Pig	Relatively inexpensive Large model size allows for greater operative surface area Good model for the uterosacral ligament	Quadruped Requires more specialized animal handlers	Kisby et al. [221] Rolland et al. [222] Baah-Dwomoh et al. [223] Tan et al. [224] Kasabwala et al. [225] Gruber et al. [226]
Sheep	Relatively inexpensive Established reproductive model Spontaneously develop prolapse Similar 3-level connective tissue paravaginally	Quadruped Requires more specialized animal handlers	Urbankova et al. [227] Jackson et al. [228] Urbankova et al. [229] Emmerson et al. [45]
Non-human primate - Squirrel monkey - Rhesus macaque	Similar reproductive physiology as human (macaque) Large fetal size in relation to pelvic outlet; more severe birth injuries Often upright, but not obligate upright, posture. Squirrel monkeys are habitual squatters and have a high risk of natural prolapse	Expensive Requires more specialized animal handlers	Otto et al. [230] Coates et al. [231] Pierce et al. [232]

It is essential to recognize the limitations of animal models and how differences in anatomy or (patho)physiology may impact translation. For example, humans are bipedal and our pelvic shape is well suited to birth a larger fetal head that accommodates our proportionally larger brain. This makes vaginal delivery more traumatic than in other species. Our erect posture and absence of a tail also have led to evolution of a complex and interconnected pelvic floor, which is not completely replicated in any animal model. Presence of a functional tail in the animal models used for research in female pelvic medicine also introduces musculature and biomechanical forces that are not present in humans. Lastly, only a few species develop spontaneous POP [230, 233–236]. Thus, many researchers focus on the intermediate outcomes, such as relevant pelvic soft tissues damage in simulated injury models or genetic manipulation that is only currently possible in the murine model (and does not represent genetics of the majority of women who develop POP.

Careful consideration should be given to the appropriate control or sham groups, blinding of researchers, and randomization techniques during preclinical investigations. When possible, control groups should be contemporaneous, rather than historical (prior experiments). Historical control groups may introduce variability that diminishes effect size. When historical controls are used due to budgetary or animal usage restrictions [237], it is important to demonstrate stability of the model with respect to the outcome(s) of interest. The above is also important to assure reproducibility of the results. In human testing, sham treatments can be an ethical challenge, particularly when studying intervention versus sham surgeries [238]. Use of sham treatments and procedures is widely accepted in animal research and these data can inform the appropriateness of a sham or control in the study design during translation.

While study outcomes are focused on on-target *efficacy*, it is also of upmost importance to evaluate on-target and off-target *safety*. For example, to evaluate the safety of a vaginally administered biologic in the USA, the Food and Drug Administration requires reporting on on-target efficacy, as well as on and off-target safety. This would include full autopsy with gross evaluation and histologic assessment of all major organ systems. Independent Pathology labs can provide blinded comprehensive tissue analysis and an unblinded report to support unbiased efficacy and safety evaluation.

### Clinical Research

#### Pilot Trials

Evaluation of the safety and efficacy of a new medical device begins with intensive preclinical (“bench”) experimentation but must, at some stage, evolve to testing in humans who suffer from the disease entity that the device was designed to treat. Although innovation is often seen as a positive development, it is currently hard to predict or establish how innovative devices improve surgical care; thus, necessitating pilot testing. Pilot studies represent a fundamental phase of the research process. The purpose of conducting a pilot study is to examine the feasibility of an approach that is intended to be used in a larger scale study. A pilot study can be used to evaluate the feasibility of recruitment, randomization, retention, assessment procedures, new methods, and implementation of the novel intervention. It is important to note that a pilot study is not a hypothesis testing study. Safety, efficacy, and effectiveness are not evaluated in a pilot. Owing to small sample size, the effect size in pilot studies is often minimal; however, these studies are valuable in that they inform sample size and power calculations for subsequent trials. A pilot study is a requisite initial step in exploring a novel intervention or an innovative application of an existent intervention. Pilot results can inform feasibility and identify modifications needed in the design of a larger, ensuing hypothesis testing study [239].

When evaluating surgical innovation, one particular challenge is the lack of standardization in the selection, measurement, and reporting of outcomes in studies of new procedures and devices, thus limiting data synthesis and the ability to accurately compare and contrast device or procedure efficacy [240]. The process of translation of devices from the laboratory to clinical practice can be seen as analogous to the process of drug development, progressing through a predictable series of unique stages, each associated with their own challenges and risks. The context is, however, very different because there are major differences in the nature of the developmental process of new drugs compared to devices in markedly different regulatory environments. The result has been that rigorous scientific evaluation of devices, especially therapeutic devices, is generally acknowledged to lag behind that of new drugs [241].

While new drugs must show substantial evidence of effectiveness and safety through clinical trials, medical device regulations in the European Union and the USA have historically focused on proof of safety as a minimum requirement. In recent years, both regulations have been updated and now demand clinical effectiveness for high-risk devices, but still allow some surgical devices to gain market approval with little or no new clinical evidence. To improve the quality of research for surgical devices, the international IDEAL collaboration, coordinated from the University of Oxford, UK, has adapted their five-stage evaluation framework (Idea, Development, Exploration, Assessment, and Long-term follow-up) to specifically provide recommendations for clinical studies on innovative devices. The central tenet of this framework is that development and evaluation can and should proceed together in an ordered and logical manner that balances innovation and safety. This collaboration aims for evaluation that results in rigorous, safe, and fast evidence gathering, using not only clinical studies but also approaches such as stakeholder consultation, modeling, and cost-effectiveness studies. The recent attention to such approaches is important, as clinical trials are expensive and the capacity for conducting these trials is limited. Ideally, only devices that have the greatest potential to improve healthcare and are aligned with the needs and beliefs of involved stakeholders are selected for clinical trials [242].

#### Randomized Controlled Clinical Trials (RCTs)

Before a new device is released onto the market, its efficacy for treating the condition for which it is designed should be proven in RCT. Once the treatment has been shown to be efficacious (and assuming that widespread complications have not become obvious during this initial evaluation), demonstrating long-term safety can be undertaken. Many adverse events may not appear in smaller initial studies with strict eligibility criteria but may be uncovered using larger diverse patient cohorts followed over a longer period of time [243].

The role of RCT in evaluating surgical interventions has been debated over the past 30 years. RCTs have an array of potential problems in evaluating surgical techniques, with most stemming from three related issues: the intervention definition, who delivers the intervention, and the treatment preferences of surgeons and patients [244]. Rising costs and inequitable access to innovative treatments, as well as recent controversies involving innovative surgical procedures elicit debate on the ethics of surgical innovation, further stifling progress.

Although the call for evidence-based practice in surgery is increasingly high on the agenda, innovation in surgery often takes place outside controlled study conditions. In most parts of the world, the clinical introduction of surgical techniques and sometimes also novel medical implants occurs with relatively little oversight and regulation. This contrasts strong regulatory and ethical requirements for the introduction of novel pharmaceuticals. There is increasing consensus that not only new drugs but also novel surgical interventions should be more thoroughly assessed before adoption into clinical practice. In general, RCTs are considered the most rigorous form of clinical research. However, most surgeons feel that the RCT format suffers from various limitations, including the perceived lack of equipoise and ethical problems related to the double-blinded design with sham surgery as a potential control. Because of these limitations and the strict format of the RCT, surgeons have been reluctant to design and implement surgical trials, also because other trial formats may be more suitable [245]. Alternative trial formats may include nonrandomized trials or cross-over trials, and prospective or retrospective cohort studies.

In the USA and UK, manufacturers and importers are required to submit reports of device related deaths, serious injuries, and malfunctions to the regulatory bodies. US hospitals and nursing homes are required to submit reports of device-related deaths and serious injuries to the manufacturer and only deaths to the FDA, but healthcare providers and consumers can submit reports voluntarily. Such passive reporting systems typically have major weaknesses but can be useful and have resulted in important public health alerts. One such example was the introduction and subsequent halt on production of transvaginal mesh kits for POP. In addition, the FDA has developed an enhanced surveillance system using several different modes, including active surveillance. This system, known as the “Medical Product Safety Network,” provides national surveillance of medical devices based on a representative subset of user facilities. Finally, when resources are available, active surveillance based on registries can also help monitor high risk surgeries and devices [244].

Across Europe and Australia, pelvic floor disorder registries have been established under the leadership of professional societies, national health services, and government-funded quality initiatives to improve patient safety and optimize device use. The Swedish National Quality Register for Gynecological Surgery (GynOp) was established in 1997 and contains data collected before, during, and after surgical treatment from close to 600,000 cases, including surgeries for POP, to date (https://www.gynop.se/home/about-gynop/). GynOp disseminates information through annual national reports and conferences for healthcare providers and hospital administrators to facilitate discussion and quality improvement. The Danish Urogynaecological Database (DugaBase) was established in 2006 to ensure high quality care for women undergoing surgeries for PFDs [246]. The French VIGI-MESH registry was established in 2016 to monitor mesh-related complications of pelvic surgeries [247]. The United Kingdom combines professional society oversight through the BSUG audit database with the NHS Pelvic Floor Registry, which tracks implants and comparators across the health system. The Netherlands requires central recording of mesh procedures through its national obstetrics and gynecology society. In Australia, the Australasian Pelvic Floor Procedure Registry (APFPR), created in response to a Senate inquiry and overseen by Monash University with federal support, integrates surgeon-reported outcomes and patient perspectives (https://apfpr.org.au/apfrp-about/). Collectively, these registries function as independent, clinician- or government-directed quality systems that allow benchmarking of surgical outcomes, early detection of safety concerns, longitudinal monitoring of device performance, and incorporation of patient-reported outcomes. The above ensures accountability, driving quality improvement, and guiding the optimization of devices and surgical practices. In the USA, American Urogynecologic Society leaders collaborated with device manufacturers, the FDA, and other professional organizations to establish the Pelvic Floor Disorders Registry (PFDR; https://www.augs.org/research/pfd-research-registry/), a collection of interrelated registries, which could meet manufacturers’ needs but also allow surgeons to track individual and aggregate outcomes for quality improvement. The PFDR was launched in 2014 with objectives of (1) collecting, storing, and analyzing clinical data related to POP treatment; (2) establishing common data elements and quality metrics; and (3) providing a framework for external stakeholders to conduct POP research. Owing to the cost of maintaining the database, this resource was closed for additional data collection in 2024. The PFDR dataset includes industry-sponsored studies, as well as two options for volunteer registry participation, the PFDR-Quality Improvement and PFDR-Research. The PFDR promotes quality improvement and national benchmarking and provides real-world comparative safety and effectiveness data for prolapse surgeries. Though currently closed for data collection, the PFDR is a model for collaboration between medical practitioners, researchers, industry, and federal agencies and, if re-opened, may allow continued benchmarking progress toward our similar goal of high-quality surgical care of women with prolapse in the USA [248].

The integration of surgical and device registries into clinical research has gained momentum toward a new era of surgical innovation, one in which every patient can contribute to global knowledge. Surgical registries harness the power of uniform data collection from varied sources to assess real-world clinical outcomes, treatment efficacy, and patient safety. These findings will enlighten the direction of surgical innovation and guide clinical and policy decision making. There are significant barriers to registry development, most notably the costly and complex nature of registry design. The availability of electronic medical record (EMR) systems is not uniform throughout the world further complicating the issue. Moreover, EMR systems variability and concerns about privacy challenge the efforts to supplant parallel, duplicate data entry with automated EMR data extraction. The ongoing goal of clinical excellence requires thorough, persistent, and honest self-assessment and transparency [249].

An ideal surveillance system for medical device safety would comprehensively collect data on adverse events across the life span of a device. Preferably, the system would be integrated into electronic health records to allow seamless identification, tracking, and real-time reporting of device-associated adverse events. Moreover, it would parse adverse events to detect substantial safety signals and underperforming devices [250].

#### Engagement with Regulatory Bodies

In the USA, the Center for Devices and Radiological Health (CDRH) of the FDA assures access to safe and effective medical devices and radiation-emitting products. CDRH provides consumers and medical professionals with evidence-based information about medical devices [251]. Industry follows transparent regulatory processes as outlined in Table 3 and Fig. 1. Most medical devices (~ 60%) in the USA are cleared through the 510(k) process versus the more rigorous premarket approval (PMA) process for higher risk devices (10%; e.g., surgical sealing device), versus 30% which are exempt from approval or clearance and go directly to market (e.g., surgical gloves) [252].

**Table 3:** Medical device risk categories

Class I	Class II	Class III
Low risk General controls With/without exemptions Premarket notification (510)k	Medium risk General controls and special controls With/without exemptions Premarket notification (510)k	High risk Class III general controls Premarket approval or Premarket notification (510)k (some devices) Investigational Device Exemption

The 510(k) premarket clearance process assumes that a new medical device demonstrates substantial equivalence to an already cleared and legally marketed device and has therefore bee deemed safe and effective. As part of the 510(k) clearance, general or special controls in the form of device registration, labeling, post-market surveillance or patient registries, and possible additional bench or animal testing data and clinical data are needed to determine substantial equivalence to a predicate device. Higher risk devices support or sustain life and require the more rigorous PMA approval process to prevent injury or unreasonable potential risk. PMA relies on clinical in addition to preclinical data. The FDA often requires post-market surveillance data on approved class III devices. Two major ways to conduct this surveillance are through Medical Device Reports (MDR) of adverse events and device malfunctions entry into the Manufacturer and User Facility Device Experience (MAUDE) Database [253] or through Sect. 522 Post-market Surveillance Studies mandated by the FDA if specific concerns or questions arise after a device has been cleared or approved [254]. Transvaginal mesh kits for POP repair were first cleared through the 510(k) process, but public health notifications were issued after reviews of MDRs in the MAUDE database in 2008 and 2010. Therefore, post-market surveillance orders were issued in 2012. By 2016, the devices were reclassified to class III, and on April 16, 2019, FDA ordered all manufacturers of transvaginal mesh kits for prolapse to stop distribution of the devices.

In the UK, the Medicines and Healthcare products Regulatory Agency (MHRA) is the official authority enforcing medical device laws [255]. General and implantable devices are also divided into low, medium, and high risk with evaluation checklists. Notably, in 2018 the UK paused the use of all surgical mesh placed transvaginally for POP and stress urinary incontinence. While the use of midurethral mesh slings for stress urinary incontinence was reinstated, mesh implantation via any route for POP is considered in complex cases like known connective tissue disorders or for failed native tissue surgery. Transvaginal mesh working groups, consisting of nominated health professionals, urged surgeon certification, improvements in mesh composition and design, careful patient selection and counseling on mesh-related specific risks and benefits preoperatively [256].

#### Patient Reported Outcomes

In the past, attention was particularly focused on anatomical outcomes following surgical intervention. In recent years, there has been a new focus on functional or composite outcomes. New international consensus recommends defining success for prolapse surgery in clinical practice as the absence of bothersome bulge symptoms. For research outcomes, success should be defined as the absence of bulge symptoms coupled with no need for repeat treatment with either pessary or surgery with a minimum of 12 months of follow-up [257].

## Conclusions and Future Directions

*“The pursuit of curiosity about the basic facts of nature has proven…to be the route by which the successful drugs and devices of modern medicine were most often discovered.”*

*Arthur Kornberg. Nobel Prize in Physiology 1959*

POP is a heterogeneous condition consequent to a myriad of predisposing, inciting, and intervening risk factors and their interactions. Given its etiologic complexity and insidious progression, currently available treatments are delayed and compensatory as they do not address the underlying pathophysiology, while preventative measures are mostly nonexistent. In fact, some of the modern therapeutic options for POP, such as pessaries, are conceptually similar to the approaches used since antiquity. To change the status quo, mechanistic investigations are necessary to promote our understanding of POP pathogenesis. The insights gained from in vitro and in vivo model systems must be thoughtfully translated into pilot clinical studies, followed by rigorous randomized controlled trials with adequate length of follow-up, and post-marketing surveillance in various populations. Ultimately, such tactics will enable a dramatic shift in clinical paradigm—instead of relying on delayed compensatory “one-size-fits-all” treatments that do not address the underlying pathophysiology, we will focus on preventing or mitigating POP through personalized medicine approaches.

Multidisciplinary research programs—adequately funded by government agencies, dedicated foundations, and industry—are mandatory for the development and ethical deployment of scientifically rational novel strategies to counteract POP. Training the next cadre of physician-scientists and full-time scientists with diverse expertise and a common interest in female pelvic medicine is an absolute prerequisite for enabling such team science efforts.

## Data Availability

N/A
